# SNP rs8099917 in Gene *IL28B* Might Be Associated with Risk of Chronic Infection by HCV but Not with Response to Treatment

**DOI:** 10.1155/2014/748606

**Published:** 2014-02-11

**Authors:** Simone Regina Souza da Silva Conde, Julius Caesar Mendes Soares Monteiro, Bruna Tereza Silva dos Santos, Nathália Karla Fonseca Filgueiras, Pedro Alves de Almeida Lins, Felipe Bonfim Freitas, Ednelza da Silva Graça, Sâmia Demachki, Marialva Tereza Ferreira de Araújo, Ricardo Ishak, Antonio Carlos Rosário Vallinoto

**Affiliations:** ^1^Laboratory of Virology, Institute of Biological Sciences, Federal University of Pará, Guamá 66075-110, Belém, PA, Brazil; ^2^School of Medicine, Institute of Health Sciences, Federal University of Pará, Praça. Camilo Salgado, No. 01, Belém, PA, Brazil; ^3^Service of Anatomic Pathology, School of Medicine, João de Barros Barreto University Hospital, Federal University of Pará, Belém, PA, Brazil

## Abstract

*Aim*. The aim of this study was to characterize the genetic profile of patients with chronic hepatitis C virus (HCV) infection relative to polymorphisms rs12979860 and rs8099917 in gene *IL28B* and the association of those polymorphisms with the response to treatment with pegylated interferon and ribavirin, performed at a reference center in Brazilian Amazonia. *Methods*. A total of 75 individuals with chronic hepatitis C and 98 healthy individuals from both genders over 18 years old were assessed. DNA samples were collected from leukocytes and subjected to real-time polymerase chain reaction to genotype polymorphisms rs12979860 and rs8099917. *Results*. Analysis of the allelic and genotypic frequencies of the investigated polymorphisms showed that both groups were in Hardy-Weinberg equilibrium; polymorphism rs12979860 exhibited no significant difference between the groups. For polymorphism rs8099917, allele T was significantly less frequent (*P* = 0.0195) among the patients (63.3%) than the controls (75.5%), and the patients were 1.7 times as likely to exhibit allele G. No difference in response to treatment was associated with SNP patterns. *Conclusion*. The results suggest a possible association of SNP rs8099917 with higher odds of chronic HCV infection but do not indicate a putative influence of the investigated SNPs on the sustained virologic response.

## 1. Introduction

Hepatitis C is considered to be an insidious disease, and its natural history has not yet been fully elucidated. According to some studies, 55 to 85% of the individuals affected by the acute form of disease remain infected for over six months and become chronic carriers [1]. In these patients, the delayed diagnosis and the fact that the infection might have remained asymptomatic over a long period of time result in advanced stages of liver cirrhosis and corresponding complications, such as bleeding esophageal varices, ascites, spontaneous bacterial peritonitis, and hepatocellular carcinoma (HCC) [2, 3].

In individuals who present with spontaneous viral clearance, the immune response is mediated by proinflammatory components of type 1 immunity [4], the type III interferon class in particular, which includes IL-29 (IFN-*λ*1), IL-28A (IFN-*λ*2), and IL-28B (IFN-*λ*3). These cytokines exhibit significant antiviral, antiproliferative, and antitumor activity and are expressed by mononuclear cells, monocyte derivatives, and dendritic cells when viral infection occurs [5]. Single-nucleotide polymorphisms (SNPs) that change the molecular expression and protein structure have been found in the genes that encode the abovementioned cytokines. SNPs have also been associated with the risk of infection and the response to treatment [4, 6, 7].

The treatment of chronic hepatitis C aims at inhibiting viral replication and eliminating the virus from the organism. The currently recommended regimen is based on the use of pegylated interferon (PEG-IFN) combined with ribavirin (RBV) for 24 to 72 weeks as a function of the virus genotype, the virologic response in the course of treatment, and the patient's tolerance [8]. The aim of the regimen is to achieve a sustained virologic response (SVR), which is defined as the absence of detectable viral RNA 24 weeks after the end of treatment [9].

Countless factors related to the host, virus, and viral kinetics have been associated with the outcome of antiviral treatment [10]. Polymorphisms in gene *IL28B*, such as rs12979860 and rs8099917, are strongly associated with the SVR [11–13].

Based on the data above and the lack of studies conducted in the Amazonian area, the aim of this study was to characterize the genetic profile of the SNPs in gene *IL28B* and their associate with SVR to treatment.

## 2. Materials and Methods

### 2.1. Case Series

The participants were recruited from January 2004 to June 2012 at the liver disease outpatient clinic of the Holy House of Mercy Foundation of Pará (Fundação Santa Casa de Misericórdia do Pará—FSCMPA), which is a regional reference in hepatology. The study protocol was approved by the institutional ethics committee (048/2011 and 108/2011).

A total of 75 consecutive patients with chronic hepatitis C from both genders (41 males and 34 females), older than 18 years old (average of 52.56 ± 10.05 years), and positive for viral RNA (PCR-RNA-HCV) were nonrandomly included. A total of 41 individuals in that sample were subjected to specific treatment with PEG-IFN combined with RBV according to the standard protocol recommended by the Brazilian Health Ministry [8]. Those cases corresponded to treatment-naive individuals, or to retreatment after the failure of conventional IFN + RBV combination therapy and had received at least one dose of the antiviral regimen (intention-to-treat analysis). Two PEG-IFN regimens were randomly indicated: PEG-IFN-*α*2b (1.5 *μ*g/kg/week) or PEG-IFN-*α*2a (180 *μ*g/kg/week) concomitant to RBV (15 mg/kg/day).

The viral kinetics of the participants were assessed according to the following criteria: (i) null response (NR), defined by the reduction of the viral RNA level less than two log by week 12 of treatment; (ii) end-of-treatment response (ETR), defined by the absence of viral RNA at week 48 of treatment; and (iii) sustained virologic response (SVR), defined by undetectable viral RNA 24 weeks after the end of treatment. Individuals coinfected with hepatitis B virus (HBV) and/or human immunodeficiency virus (HIV) were excluded.

The control group comprised 98 healthy individuals from both genders (39 males and 59 females) who were older than 18 years old (average of 37.78 ± 15.09 years) and seronegative for HCV, HBV, and HIV.

In the present study we were careful to include only individuals from the same ethnic background residents in urban or rural area of the state of Para. Caucasians, Amerindians, and Afro-Brazilian communities were excluded in order to avoid and reduce biases.

### 2.2. Sample Collection

After the participants expressed their agreement to participate in the study and signed an informed consent form, the epidemiological, clinical, laboratory, and histopathological data in their clinical records were entered into a standard form. Samples of peripheral blood (5 mL) were collected for the extraction of total DNA from leukocytes using a modification of the phenol-chloroform method described by Sambrook and Russel [14], followed by cell lysis, protein precipitation, and DNA precipitation and hydration. Samples of viral RNA were obtained based on the extraction of total RNA from the plasma using the Total RNA Purification Kit (NORGEN, Biotek Corporation, Canada).

### 2.3. Genotyping

Polymorphisms rs12979860 (C > T) and rs8099917 (T > G) in gene *IL28B* were genotyped by real-time PCR (qPCR) using a Step One PLUS Sequence Detector (Applied Biosystems, Foster City, CA, USA).

Each polymorphism assay contained one pair of primers and one pair of probes, and each allele of the polymorphisms was labeled with VIC or FAM. The rs12979860 target sequence was sent to the Applied Biosystems company, which designed the following primer and probe sequences: 5′-GCC TGT CGT GTA CTG AAC CA-3′ (forward), 5′-GCG CGG AGT TGC AAT TCA AC-3′ (reverse), 5′VIC-TGG TTC GCG CTT C-3′ (allele C probe), and 5′FAM-CTG GTT CAC GCC TTC-3′ (allele T probe). For polymorphism rs8099917, an assay predesigned by Applied Biosystems and identified as C_11710096_10 was used.

Each reaction included [1X] TaqMan Universal PCR Master Mix [2X], [1X] TaqMan Assay [20X], and 20 ng of DNA in a total volume of 10 *μ*L. The following parameters were used in amplification and allele detection: one cycle at 60°C for 30 seconds, followed by one cycle at 95°C for 10 minutes and 50 cycles at 92°C for 30 seconds and 60°C for 90 seconds.

### 2.4. Viral Load, Genotyping, and Histopathological Examination

The viral load and genotyping tests were performed at Central Laboratory using the qualitative and quantitative PCR-HCV-RNA method (Roche-COBAS AMPLICOR Hepatitis C Virus (HCV) Test, version 2.0 v). For the purpose of analysis, the viral load values were dichotomized as low (<600,000 IU/mL) or high (>600,000 IU/mL). The histopathological assessment followed the French Metavir classification [15].

### 2.5. Statistical Analysis

Descriptive and inferential analyses were performed as a function of the normality of the data. Parametric ANOVA and Student's *t*-test were used for quantitative data, and the nonparametric chi-square test (with the Yates correction), Fisher's exact test, and *G*-test (with Williams' correction) were used for qualitative nominal or ordinal data. The genotypic and allelic frequencies found were assessed using the Hardy-Weinberg equilibrium.

The degree of association between genotypes and viral infection was measured using the odds ratio (OR) and corresponding 95% confidence intervals (CI). The analyses were performed using the software Epi Info 3.5.3 [16] and BioEstat 5.3 [17], with alpha ≤ 5% established as the level to reject the null hypothesis.

## 3. Results

Clinical and laboratory assessment of the stage of hepatitis showed that 76% (57/75) of the participants did not exhibit liver cirrhosis, while 24% (18/75) did.

The viral genotype was established in 70 participants, with genotype 1 in 77.1% (54/70) of the cases and genotype 3 in 22.9% (16/70). Among the participants subjected to treatment, 80.5% (33/41) exhibited genotype 1, and 19.5% (8/41) exhibited genotype 3.

The viral load was measured in 61 participants and was less than 600,00 IU/mL in 63.9% (39/61) of the cases.

Liver biopsy was performed in 59 participants; histopathological analysis showed that 61% (36/59) of the biopsies exhibited activity periportal or periseptal scores of zero to one, and 59.3% exhibited fibrosis scores of two to four.

Analysis of the SNP rs12979860 allelic and genotypic frequencies showed that both groups (patients and controls) were in Hardy-Weinberg equilibrium (Table 1). A tendency (*P* = 0.0813) to a higher frequency of allele T was found in the group of patients compared to the controls, but no significant difference in the genotypic frequency (*P* = 0.1865) was found between the two groups.

Analysis of the polymorphism rs8099917 allelic frequency (Table 1) showed that both groups (patients and controls) were in Hardy-Weinberg equilibrium. Allele T was less frequent in the group of patients (63.3%) compared to the controls (75.5%), and the probability of patients exhibiting allele G was 1.7-fold higher than in the controls. These differences were statistically significant (*P* = 0.0195). In addition, a tendency (*P* = 0.0600) to a higher proportion of genotype TT (*P* = 0.0413) was found in the group of controls (58.2%) compared to the patients (41.3%), whereas the frequency of genotype GG was twice as high among the patients (14.7%) compared to the controls (7.1%).

Among all patients treated with PEG-IFN + RBV, 61% (25/41) were undergoing their first treatment for hepatitis C, and 39% (16/41) were being retreated due to the failure of treatment with conventional IFN.

**Figure 1:**
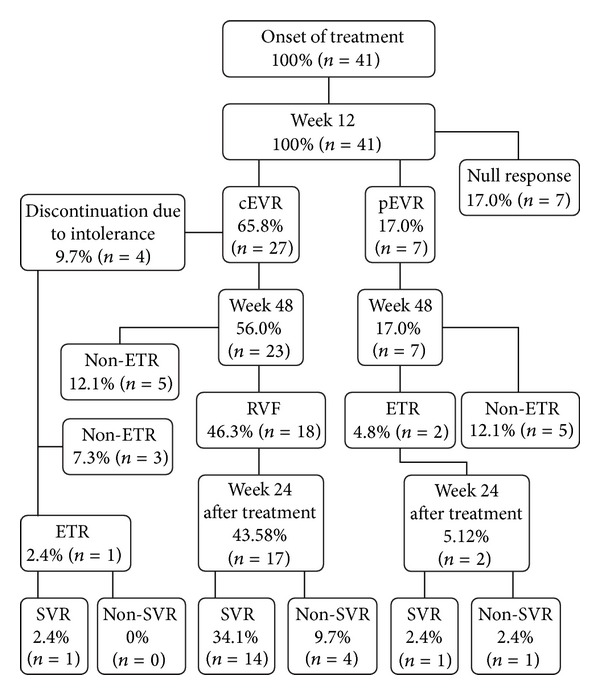
Follow-up of the treatment of individuals with chronic hepatitis C treated with PEG-IFN and RBV at FSCMPA from January 2004 to January 2012. cEVR: complete early virologic response; pEVR: partial early virologic response; ERT: end-of-treatment response; SVR: sustained virologic response.

Intention-to-treat analysis resulted in 51.21% of ERT (21/41) and 39% of SVR (16/41). Treatment discontinuance due to NR or non-ERT occurred in 41.4% of the cases (17/41), and intolerance to side effects occurred in 9.7% (4/41) (Figure 1). Neither SNP rs12979860 nor rs8099917 exhibited a statistically significant correlation with SVR (Table 2).

## 4. Discussion

In this study, no significant difference was found in the genotype and allele proportions of SNP rs12979860, possibly due to the small size of the sample of individuals with hepatitis C. Nevertheless, genotype CT predominated among the patients (49.3%), which agrees with the results reported by Ge et al. [11] for North-American patients with hepatitis C, whereby genotype CT was found in 49.7% of the individuals of European ancestry, 47.6% of African-Americans, and 46.7% of Hispanics. In addition, Bochud et al. [7] and Lunge et al. [18] found a higher prevalence of genotype CT in Caucasian Europeans (49%) and Brazilians (60.6%) infected by HIV-1, respectively. In contrast, the prevalence of genotype CC is high in the Brazilian (51.4%), Taiwanese (89.8%), South-Korean (87.7%), and Japanese (76.8%) populations [3, 6, 19, 20]. Those differences might be attributed to the ethnic background of each particular population or to the selective pressure exerted by environmental factors.

The allelic distribution of polymorphism rs12979860 in the group of patients was similar to the distribution described by Venegas et al. [21] relative to individuals treated for HCV in Chile. Additionally, Ge et al. [11] found a high frequency of allele T in Afro-Americans (60.5%), differing from other human populations [19, 20, 22].

Analysis of the SNP rs8099917 genotypes revealed a tendency (*P* = 0.06) to a higher frequency of genotype TT among the controls and GT among the patients. The higher frequency of the heterozygous genotype GT and homozygous genotype GG among the patients agrees with the results reported by Venegas et al. [21]. However, it disagrees with the findings of other studies conducted with individuals infected by HCV, according to which the frequency of genotype TT was higher than any other genotype in Caucasian Europeans [22, 23], Japanese [3], and Taiwanese [6].

For polymorphism rs8099917, the distribution of allele G exhibited a significant difference between the groups, being higher among the patients than the controls, and the presence of that allele was associated with 1.8 greater odds of becoming chronically infected by HCV. The results of this study point to a possible association between the presence of that SNP and greater odds of susceptibility to chronic infection by HCV. However, to confirm this hypothesis it would be necessary to analyze a group of individuals who were exposed to HCV, but subsequently cleared the virus. Only one study, by Venegas et al. [21], has reported a high frequency of that allele, while in other studies, it varied from 5.2% [6] to 25.5% [24]. Nevertheless, the variation of the allelic frequency of this SNP among different human populations suggests that the presence of an unfavorable genotype (GG) alone does not account for the susceptibility to infection, and other genetic factors not investigated in this present study might also be involved in that phenomenon.

The rate of SVR exhibited by the treated patients was 39%, which is lower than the rate found in the previous studies by Hadziyannis et al. [25] using PEG-IFN alfa-2b, which was 63%. However, it is similar to the rates found in studies conducted with patients with only genotype 1 of HCV (39.8–46%), which agrees with the predominance of that genotype among the group of treated patients in the present study [10, 26, 27].

## 5. Conclusions

We did not find a significant association of SNPs rs12979860 and rs8099917 with SVR, thus disagreeing with studies that found an association between genotype CC (rs12979860) and SVR in individuals with genotype 1 [10, 11, 27], 2, and 3 [13], as well as between genotype TT (rs8099917) and SVR in individuals with genotype 1 [12].

## Figures and Tables

**Table 1:** Genotypic and allelic frequencies of SNP rs12979860 and rs8099917 in gene *IL28B* in individuals with chronic hepatitis C virus infection and the control group, Belém, 2012.

SNPs	Patients *n* (%)	Controls *n* (%)	*P* ^a^	OR (CI 95%)	P^b^
rs12979860					
Genotypes					
* *CC	18 (24.0)	34 (36.7)	0.1865	0.5944 (0.3031–1.1659)	0.1761
* *CT	37 (49.3)	47 (48.0)		1.0566 (0.5789–1.9283)	0.9795
* *TT	20 (26.7)	17 (17.3)		1.7326 (0.8336–3.6013)	0.1955
Alleles					
* *C	73 (48.7)	115 (58.7)	0.0813	0.6678 (0.4352–1.0247)	0.0813
* *T	77 (51.3)	81 (41.3)			

rs8099917					
Genotypes					
* *GG	11 (14.7)	07 (7.1)	0.0600	1.2344 (0.8219–6.0745)	0.1754
* *GT	33 (44.0)	34 (34.7)		1.4790 (0.7979–2.7416)	0.2767
* *TT	31 (41.3)	57 (58.2)		0.5068 (0.2753–0.9329)	0.0413
Alleles					
* *G	55 (36.7)	48 (24.5)	0.0195	1.7851 (1.1212–2.8420)	0.0195
* *T	95 (63.3)	148 (75.5)			

^a^Chi-square; ^b^odds ratio.

**Table 2:** Association of SNPs rs12979860 and rs8099917 with sustained virologic response in individuals with chronic hepatitis C treated with PEG-IFN and RBV.

SNPs	Type of response		*N*	OR (CI 95%)	P*	P^a,b^
	SVR	Non-SVR + Non-ETR + NR				
rs12979860						
Genotypes						
* *CC	08 (61.5)	05 (38.5)	13	4.00 (1.0004–0.0950)	0.0950	0.1454^a^
* *CT	06 (30.0)	14 (70.0)	20	0.47 (0.1306–1.7020)	0.4033	
* *TT	02 (25.0)	06 (75.0)	08	0.45 (0.0792–2.5848)	0.6153	
Alleles						
* *C	22 (46.8)	24 (53.2)	47	2.38 (0.9395–6.0461)	0.1055	0.1055^b^
* *T	10 (27.8)	26 (72.2)	36			

rs8099917						
Genotypes						
* *GG	01 (25.0)	03 (75.0)	04	0.49 (0.0463–5.1595)	0.9475	0.5908^a^
* *GT	06 (33.3)	12 (66.7)	18	0.65 (0.1806–2.3393)	0.7351	
* *TT	09 (47.4)	10 (52.6)	19	1.93 (0.5410–6.8755)	0.4859	
Alleles						
* *G	8 (30.8)	18 (59.2)	26	0.59 (0.2209–1.5897)	0.4232	0.4232^b^
* *T	24 (42.9)	32 (57.1)	56			

*OR (CC*  *versus*  *CT*  *versus*  *TT). ^a^ *G*-test; ^b^chi-square test.

SVR: sustained virologic response; ETR: end-of-treatment response; NR: null response.
